# Single cell analysis of clonal architecture in acute myeloid leukaemia

**DOI:** 10.1038/s41375-018-0319-2

**Published:** 2018-12-19

**Authors:** Nicola Potter, Farideh Miraki-Moud, Luca Ermini, Ian Titley, Gowri Vijayaraghavan, Elli Papaemmanuil, Peter Campbell, John Gribben, David Taussig, Mel Greaves

**Affiliations:** 1 Centre for Evolution and Cancer, The Institute of Cancer Research, London, UK; 2 Barts Cancer Institute, Queen Mary University of London, London, UK; 3 Memorial Sloan Kettering Cancer Center, New York, USA; 4 Wellcome Sanger Institute, Hinxton, UK; 5 Royal Marsden Hospital, Sutton, UK

**Keywords:** Cancer stem cells, Cancer genomics

## Abstract

We used single cell Q-PCR on a micro-fluidic platform (Fluidigm) to analyse clonal, genetic architecture and phylogeny in acute myeloid leukaemia (AML) using selected mutations. Ten cases of *NPM1*c mutant AML were screened for 111 mutations that are recurrent in AML and cancer. Clonal architectures were relatively simple with one to six sub-clones and were branching in some, but not all, patients. *NPM1* mutations were secondary or sub-clonal to other driver mutations (*DNM3TA, TET2, WT1* and *IDH2*) in all cases. In three of the ten cases, single cell analysis of enriched CD34^+^/CD33^−^ cells revealed a putative pre-leukaemic sub-clone, undetectable in the bulk CD33^+^ population that had one or more driver mutations but lacked *NPM1*c. Cells from all cases were transplanted into NSG mice and in most (8/10), more than one sub-clone (#2-5 sub-clones) transplanted. However, the dominant regenerating sub-clone in 9/10 cases was *NPM1*^+^ and this sub-clone was either dominant or minor in the diagnostic sample from which it was derived. This study provides further evidence, at the single cell level, for genetic variegation in sub-clones and stem cells in acute leukaemia and demonstrates both a preferential order of mutation accrual and parallel evolution of sub-clones.

## Introduction

Although almost all cancers originate in a single cell, the sequential acquisition of necessary additional mutations fuels sub-clonal diversity which is then a substrate for positive or negative selection within the tissue ecosystems and with therapy [1, 2]. This process frequently results in complex cell population structures and highly variegated genetics [3–5].

The genomics of AML have been described in considerable detail, revealing multiple sub-types [6, 7] and sequential transition between clinically silent pre-leukaemia and overt disease [8, 9]. Sub-clonal architectures in diagnostic samples have been inferred from allele burdens [10] and appear to be relatively simple compared to that observed in many common cancers [11, 12].

The extent of sub-clonal diversity and phylogenetic architecture is, however, best derived from single cell analysis. This is challenging however in terms of accuracy and depth, but has proven illuminating in some solid tumours [13] and ALL [3].

Only a few studies to date have reported single cell genetics and inferred sub-clonal phylogenies in AML. Paguirigan et al. [14] used single cell, multiplexed Q-PCR to investigate patterns of segregation of two concurrent mutations in AML–*FLT3*^−^ITD and *NPM1*c mutations. The data revealed significantly more sub-clonal diversity than could be inferred from analysis of the bulk population. Klco et al. [15] fractionated immuno-phenotypically distinct cell populations from a patient with AML and sequenced the amplified DNA from single cells for ten known mutations. From these data, they could infer a branching sub-clonal architecture. Jan et al. used a Q-PCR assay on colonies derived from sorted single cells derived from two cases of AML and were able to infer an ordered sequence of mutations [8]. Quek et al. screened single cells for targeted mutations in immunophenotypically-defined subsets and identified putative clonal sequences and mutation order in six cases [16].

In our previous studies in ALL, we used multi-colour FISH or multi-plexed Q-PCR in a micro-fluidic platform (Fluidigm) to detect sub-clonal variegation and clonal architecture [3, 17]. In this study, we sought to replicate our observations on ALL for AML, selecting the subset of cases with *NPM1*c mutations. *NPM1c*^+^ cases constitute around 27% of adult AML with a variable but overall intermediate risk [7]. The questions posed included the extent of sub-clonal complexity that was discernible, sequential order of mutations and whether stem cells or leukaemia propagating cells, assayed by xeno-transplantation, were genetically variable.

## Materials and methods

### Sample cohort

A total of ten well-characterised *NPM1* mutant AML samples [18] were selected for further study according to engraftment potential. Blood and marrow samples were collected from patients with AML after written informed consent at St Bartholomew’s Hospital. The protocol was approved by the East London and City Research Ethics Committee. All studies comply with the rules of the revised Helsinki protocol. These had all been found to successfully transplant in NOD/SCID mice [18]. This selection criterion may have biased our analysis towards poorer prognosis cases [7, 18]. Available peripheral blood was collected prior to treatment at presentation (*n* = 10) and from matched relapse (*n* = 3). Mononuclear cells were obtained by density gradient centrifugation. Details of the patient samples are listed (Table 1).

**Table 1:** Patient information including treatment details, tracked mutations and sub-clone indications

Patient	Patient treatment details	Tracked mutations	No. clones CD33+CD3− fraction	No. clones CD34+CD33− fraction	No. clones in xenografts	Total detected clones
1	Died post induction	*TET2 x2 (one not tracked), DNMT3A, NPM1,FLT3-ITD*	4	3	3	5
2	Refractory to primary induction	*WT1, IDH2,NRAS, NPM1,GATA1*	3	2	2	3
3	Relapsed	*WT1,NPM1,FLT3 x2*	6	5	2	6
4	Received palliative chemotherapy	*DNMT3A,TET2,CBL,FLT3-ITD, NPM1*	2	2	4	4
5	Not offered chemotherapy as had co-existing colon cancer	*DNMT3A,TET2,ZRSR2,NPM1, FLT3-ITD, PTPN11, NF1*	4	3	5	6
6	Responded to induction; remains in remission	*DNMT3A (no tracked), TET2,NPM1*	2	2	2	2
7	Went into remission; developed therapy related MDS	*DNMT3A, TET2 x2, NPM1,FLT3, CTNNA1 (not tracked)*	2	2	1	4
8 (diagnostic sample)	Relapsed	*DNMT3A,MLL5,NPM1, FLT3-ITD,GATA2, TET2*	1	2	2	3
8 (relapse sample)	Died	*DNMT3A,MLL5,NPM1,FLT3-ITD,GATA2, TET2*	1	1	1	2
9 (diagnostic sample)	Relapsed	*WT1,NPM1,FLT3-ITD, MLL3 and UTY (not tracked)*	1	1	1	1
9 (relapse sample)	Died	*WT1,NPM1,FLT3-ITD, MLL3 and UTY (not tracked)*	1 (bulk cells)	–	1	1
10 (diagnostic sample)	Relapsed	*DNMT3A,NPM1, TP53, FLT3 x2 (neither tracked)*	2	3	Did not engraft	3
10 (relapse sample)	Died	*DNMT3A,NPM1, TP53 FLT3 x2 (neither tracked)*	3 (bulk cells)	–	2	3

FACS cell sorting according to immunophenotype details can be found in Supplementary Information.

### Mutation analysis

A targeted screening approach investigating 111 genes (Table 2) was used to identify mutations and DNA coding region alterations in each *NPM1*c AML as previously described [7] that could potentially be tracked in single cells. The analysis is based on variants that can be classified as recurrent driver mutations, using widely accepted genetic criteria. These included non-synonymous base substitutions and small (<200-bp) insertions or deletions (indels). Table 3 lists the probes used for mutant versus wild type sequences and PCR primers.

**Table 2:** List of 111 genes commonly mutated in AML and cancer screened using targeted NGS

Symbol	Ensembl ID	NCBI	Position	Symbol	Ensembl ID	NCBI	Position	Symbol	Ensembl ID	NCBI	Position
ABCA12	ENSG00000144452	26154	2q34	GATA2	ENSG00000179348	2624	3q21.3	NUP98	ENSG00000110713	4928	11p15.4
ABL1	ENSG00000097007	25	9q34.1	GNAS	ENSG00000087460	2778	20q13.3	OCA2	ENSG00000104044	4948	15q12-q13.1
ACTR5	ENSG00000101442	79913	20q11.23	HIPK2	ENSG00000064393	28996	7q34	PDGFRA	ENSG00000134853	5156	4q12
ARHGAP26	ENSG00000145819	23092	5q31	HRAS	ENSG00000174775	3265	11p15.5	PHF12	ENSG00000109118	57649	17q11.2
ASXL1	ENSG00000171456	171023	20q11.1	HMGA2	ENSG00000149948	8091	12q15	PHF6	ENSG00000156531	84295	Xq26.2
ATRX	ENSG00000085224	546	Xq21.1	IDH1	ENSG00000138413	3417	2q33.3	PKP3	ENSG00000184363	11187	11p15
ATXN7L1	ENSG00000146776	222255	7q22.3	IDH2	ENSG00000182054	3418	15q26.1	PRDX2	ENSG00000167815	7001	19p13.2
BCOR	ENSG00000183337	54880	Xp11.14	IKZF1	ENSG00000185811	10320	7p13	PRPF40B	ENSG00000110844	25766	12q13.12
BRAF	ENSG00000157764	673	7q34	INVS	ENSG00000119509	27130	9q31	PTEN	ENSG00000171862	5728	10q23.3
CBL	ENSG00000110395	867	11q23.3	IRF1	ENSG00000125347	3659	5q31.1	PTPN11	ENSG00000179295	5781	12q24.1
CBLB	ENSG00000114423	868	3q13.11	JAK2	ENSG00000096968	3717	9p24	RAD21	ENSG00000164754	5885	8q24.11
CBLC	ENSG00000142273	23624	19q13.2	JAK3	ENSG00000105639	3718	19p13.1	RAD50	ENSG00000113522	10111	5q31.1
CD101	ENSG00000134256	9398	1p13	KDM2B	ENSG00000089094	84678	12q24.31	RB1	ENSG00000139687	5925	13q14
CDH1	ENSG00000039068	999	16q22.1	KDM5A	ENSG00000073614	5927	12p13.33	RINT1	ENSG00000135249	60561	7q22.3
CDKN1B	ENSG00000111276	1027	12p13.1	KDM6A	ENSG00000147050	7403	Xp11.2	RORC	ENSG00000143365	6097	1q21
CDKN2A	ENSG00000147889	1029	9p21	KIT	ENSG00000157404	3815	4q12	RUNX1	ENSG00000159216	861	21q22.3
CDKN2B	ENSG00000147883	1030	9p21.3	KRAS	ENSG00000133703	3845	12p12.1	RUNX1T1	ENSG00000079102	862	8q22
CEBPA	ENSG00000245848	1050	19q13.1	LCORL	ENSG00000178177	254251	4p15.31	SF1	ENSG00000168066	7536	11q13.1
CHGA	ENSG00000100604	1113	14q32	LILRA3	ENSG00000170866	11026	19q13.4	SF3A1	ENSG00000099995	10291	22q12.2
CREBBP	ENSG00000005339	1387	16p13.3	MAP2K5	ENSG00000137764	5607	15q23	SF3B1	ENSG00000115524	23451	2q33.1
CSF1R	ENSG00000182578	1436	5q32	MET	ENSG00000105976	4233	7q31	SH2B3	ENSG00000111252	10019	12q24.12
CSF2	ENSG00000164400	1437	5q31.1	MLL	ENSG00000118058	4297	11q23	SOCS1	ENSG00000185338	8651	16p13.13
CTNNA1	ENSG00000044115	1495	5q31	MLL2	ENSG00000167548	8085	12q12	SPI1	ENSG00000066336	6688	11p11.2
CUX1	ENSG00000160967	1523	7q22.1	MLL3	ENSG00000055609	58508	7q36.1	SRPK2	ENSG00000135250	6733	7q22.3
DDX18	ENSG00000088205	8886	2q14.1	MLL5	ENSG00000005483	55904	7q22.3	SRSF2	ENSG00000161547	6427	17q25.1
DNMT1	ENSG00000130816	1786	19p13.2	MMD2	ENSG00000136297	221938	7p22.1	STAG2	ENSG00000101972	10735	Xq25
DNMT3A	ENSG00000119772	1788	2p23	MN1	ENSG00000169184	4330	22q12.1	STK17B	ENSG00000081320	9262	2q32.3
EGFR	ENSG00000146648	1956	7p12	MPL	ENSG00000117400	4352	1p34.2	TCF4	ENSG00000196628	6925	18q21.2
ELF1	ENSG00000120690	1997	13q14.11	MTAP	ENSG00000099810	4507	9p21.3	TET1	ENSG00000138336	80312	10q21.3
EP300	ENSG00000100393	2033	22q13	MYC	ENSG00000136997	4609	8q24.21	TET2	ENSG00000168769	54790	4q24
ERG	ENSG00000157554	2078	21q22.2	NF1	ENSG00000196712	4763	17q11.2	TP53	ENSG00000141510	7157	17p13.1
ETV6	ENSG00000139083	2120	12p13.2	NLRP1	ENSG00000091592	22861	17p13.2	U2AF1	ENSG00000160201	7307	21q22.3
MECOM	ENSG00000085276	2122	3q26	NOTCH1	ENSG00000148400	4851	9q34.3	U2AF2	ENSG00000063244	11338	19q13.42
EZH2	ENSG00000106462	2146	7q35-36	NPM1	ENSG00000181163	4869	5q35	WT1	ENSG00000184937	7490	1p13
FAM175B	ENSG00000165660	23172	10q26.13	NR5A1	ENSG00000136931	2516	9q33	ZEB2	ENSG00000169554	9839	2q22.3
FBXW7	ENSG00000109670	55294	4q31.3	NRAS	ENSG00000213281	4893	1p13.2	ZRSR2	ENSG00000169249	8233	Xp22.1
FLT3	ENSG00000122025	2322	13q12	NRD1	ENSG00000078618	4898	1p32.2-p32.1				
GATA1	ENSG00000102145	2623	Xp11.23	NSD1	ENSG00000165671	64324	5q35.2

**Table 3:** Patient specific allelic discrimination Q-PCR assay information

Gene Reference	Mutation	Patient	Probe-wild type seq-VIC lablelled	Probe-mutant seq-FAM lablelled	Forward primer	Reverse primer
CBL	p.G413D	Patient 4	AGGAATCAGAAGGTCAG	AGGAATCAGAAGATCAG	TGCATCTGTTACTATCTTTTGCTTCTTC	ATTTCACATCGGCAGAAAGGA
DNMT3A	p.R882C	Patient 1	CCAAGCGGCTCAT	CCAAGCAGCTCAT	CCGGCCCAGCAGTCTCT	CAGTCCACTATACTGACGTCTCCAA
DNMT3A	p.M682fs*23	Patient 4	N/A	CGACGTACATATCTTC	CCCCACAGCATGGACATACA	CATCACGGTGGGCATGGT
DNMT3A	p.R882H	Patient 5, 7, 8, 10	CCAAGCGGCTCAT	CCAAGTGGCTCATG	CCGGCCCAGCAGTCTCT	TGGTTTCCCAGTCCACTATACTGA
FLT3	p.D835E	Patient 7	ACTCATGATATCTCG	TCACTCATGATCTCTCGA	GCCCCTGACAACATAGTTGGA	GTGGTGAAGATATGTGACTTTGGATT
FLT3	p.M664I	Patient 3	CTGGGTCATCATCT	CTGGGTCATTATCT	CCCCAGCAGGTTCACAATATTC	AAGAGAGGCACTCATGTCAGAACTC
FLT3	p.N841K	Patient 3	CTGACAACATAGTTGGAA	CTGACAACATATTTGG	AAATAAGTAGGAAATAGCAGCCTCACA	GGATTGGCTCGAGATATCATGAGT
GATA1	p.P38L	Patient 2	CCTCTGGGCCTGAG	TGGGCTTGAGGGC	GTGTCCTCCACACCAGAATCAG	GAGGAAGCTGCTGCATCCA
GATA2	p.N402S	Patient 8	TGGACTTGTTGGACAT	TCTTCTTGGACTTGCTG	TTTGACAGCTCCTCGAAGCA	CAGGCCACTGACCATGAAGA
IDH2	p.R140Q	Patient 2	CCAGGATGTTCCGGAT	CCAGGATGTTCTGGAT	GGGCTCCCGGAAGACAGT	TGTGGAAAAGTCCCAATGGAA
MLL5	p.S556N	Patient 8	AACTCCTATTAGTAATGAAG	AACTCCTATTAATAATGAAG	CATTTTTCAGGAACCAGATTTTATTG	CATCTTCCTTTTCCTTTCTGCAA
NF1	p.S2243fs*14	Patient 5	N/A	ATATAATCCATTCCCTGCAACC	TCTTTTAATTGCAGATTTGCATTCC	GCTAATACACCCAAAGACAACAAGAG
NPM1-B	p.W288fs*12	Patient 1, 5, 9	N/A	TTCCAGGCTATTCAAG	ATGTCTATGAAGTGTTGTGGTTCCTT	TCCTCCACTGCCAGACAGAGA
NPM1-A	p.W288fs*12	Patient 3, 4, 6, 7, 8, 10	N/A	AAGATCTCTGTCTGGCAGTG	TGTCTATGAAGTGTTGTGGTTCCTTAA	CTGTTACAGAAATGAAATAAGACGGAAA
NPM1-D	p.W288fs*12	Patient 2	N/A	TTCAAGATCTCTGCCTGGC	TGTCTATGAAGTGTTGTGGTTCCTTAA	CTGTTACAGAAATGAAATAAGACGGAAA
NRAS	p.G13D	Patient 2	CCAACACCACCTGC	CCAACATCACCTGCT	CTGGATTGTCAGTGCGCTTTT	TTGCTGGTGTGAAATGACTGAGT
PTPN11	p.E76G	Patient 5	CCACTTTGGCTGAGT	CCACTTTGGCTGGGTT	CACCCACATCAAGATTCAGAACAC	CCCGTGATGTTCCATGTAATACTG
TET2	p.L1469fs*9	Patient 7	N/A	CGACAAAGGAAAACTA	TGTTAGCAGAGCCAGTCAAGACTT	TCCAGGGAGGAAAGCTTTTCA
TET2	p.Q1624*	Patient 7	TTTGAATCAGAATACCCAAT	TGGGCTTTTGAATTAGAATA	CTTCTAATCCCATGAACCCTTACC	CCACTGATAGGTTTCCATTGCA
TET2	p.R544*	Patient 1	CTGAAGGGTCGAGACAA	CTGAAGGGTTGAGACA	GCCAGCAGTTGATGAGAAACAA	GGCACAAGATCTCGTGTTTGC
TET2	p.S1369*	Patient 4	CCGTCCATTCTCAGG	CCGTCCATTCTGAGG	GCCGTCTGGGTCTGAAGGA	ACAGAAGTCCAAACATGCAGTGA
TET2	p.V1417F	Patient 5, 8	CAGCTTCACGTTCTG	AGCTTCACTTTCTGCCT	TGGAGGAAAACCTGAGGATGA	GAGCTTCCACACTCCCAAACTC
TET2	p.C1374Y	Patient 6	TCTCAAGGAAACCCCAG	TCTCAAGGAAACGCCAG	CAAAAATGTTTGCTCAGGACACA	TCGTGAACCCAACTCTTCTAACTG
TP53	p.R248Q	Patient 10	ATGGGCCTCCGGTT	ATGGGCCTCTGGTT	GGCTCCTGACCTGGAGTCTTC	TGACTGTACCACCATCCACTACAA
WT1	p.A382fs*4	Patient 9	N/A	AGATGCCGACCGACC	GCCTGGTAAGCACACATGA	TGGAGTAGCCCCGACTCTTG
WT1	p.Y402	Patient 2	ACAGCTTAAAATATCTC	ACAGCTTAAACTATCTC	TCCTGCTGTGCATCTGTAAGTG	TGCTTACCCAGGCTGCAATAA
WT1	p.L349fs*26	Patient 3	N/A	CGCAGAGATGGGC	CCGTGCGTGTGTATTCTGTATTG	ACAGGGTACGAGAGCGATAACC
ZRSR2	p.Y274*	Patient 5	TGTATATGTTCAGTACCAGTC	CAATGTATATGTTCAGTAACA	CTAGGTCAGCTGCAATTTGGAA	ACAAATCAGGAAGACACAAG

### Sequencing data

For the targeted mutation screening of each leukaemia, two populations of interest were stained and sorted as described in Supplementary Information and DNA extracted (Qiagen® DNA blood kit according to manufacturers’ instructions): peripheral blood T-cells (CD3^+^/CD33^−^) (as a control) and mononuclear blast cells (CD3^−^/CD33^+^). The latter had <1% CD34^+^ cells and we refer to this population as CD34^−^.

For details of library preparation, sequencing, alignment and analysis, please refer to Supplementary Information.

### Xeno-transplantation

NOD/SCID (Il2rg^−/−^) mice (Jackson Laboratory, Bar Harbor, ME) were injected intravenously (3 mice per AML sample) with 9–10 million AML cells after T-cell depletion by Easysep T-cell enrichment cocktail (Stem Cell Technologies). Mice were bled by tail veins at 12–14 weeks and blood leucocytes investigated by FACS (as described in Supplementary Information and Supplementary Figs. 2 and 3) using anti-human and anti-mouse CD45 antibodies to determine the percentage of leukaemic cell engraftment. For details of how successful/undetectable/minimal grafts were managed and serial transplantations were carried out, please refer to Supplementary Information.

### Single cell sorting and multiplex Q-PCR analysis

Single cell sorting was carried out (see Supplementary Information and Supplementary Fig. 1) according to our established published Q-PCR single cell (Fluidigm) protocol [17]. Briefly, from each case single AML cells (either CD33^+^/CD34^−^/CD3^−^ (blast population), CD3^+^/CD33^−^ (internal control), CD34^+^/CD33^−^ (putative stem cell), CD45^+^ (human cells post-transplant) or cord blood cells (normal diploid control) were sorted into individual wells of a 96 well plate, lysed and DNA target amplification completed for regions of interest encompassing patient specific mutations or DNA alterations. Allelic discrimination Q-PCR assays were designed specifically for each mutation in every patient. Standard Q-PCR assays targeting unique *FLT3*^*−*^*ITDs* were designed for each positive patient. Genes targeted in each case are listed in Table 1. The *ß2M* locus, located in a diploid region of the genome, was used as a control. Q-PCR completed using the 48 × 48 dynamic array and the BioMark™ HD from Fluidigm.

Several approaches were adopted during this experiment to optimise and confirm the presence of a single cell and ensure all assays performed efficiently under experimental conditions [17]; a brief description can be found in Supplementary Information and Supplementary Fig. 4.

### Maximum parsimony

Maximum parsimony searches for sub-clonal phylogenies were conducted using heuristic searches as previously described [17]; a brief description can be found in Supplementary Information.

## Results

Our targeted exomic screening approach identified a number of common or recurrent driver SNV mutations in each patient’s diagnostic sample (Table 1); similar to those previously described for *NPM1c* AML [7, 19–21]. Five of the ten cases had both *DNM3TA* and *TET2* mutations, reflecting the selection of driver mutations that cooperate to confer fitness advantage of haemopoietic stem cells [22]. Allele frequencies varied greatly suggested that many mutations were probably sub-clonally distributed.

Individual cells sorted as CD34^+^/CD33^−^ or CD33^+^/CD3^−^ were assayed by multiplex Q-PCR for each driver mutation identified in that patient’s sample. We similarly assessed individual cells (unsorted) from NSG mice in which T-cell depleted AML cells from each patient had been transplanted. From those single cell data, we are able to infer a probable clonal phylogeny for each case with genetically distinct sub-clones, the immunophenotype and the clonal derivation of leukaemia that regenerated in NSG mice. We take the latter as a read-out of sub-clones with self-renewal or stem cell activity.

### Clonal architectures

**Fig. 1:**
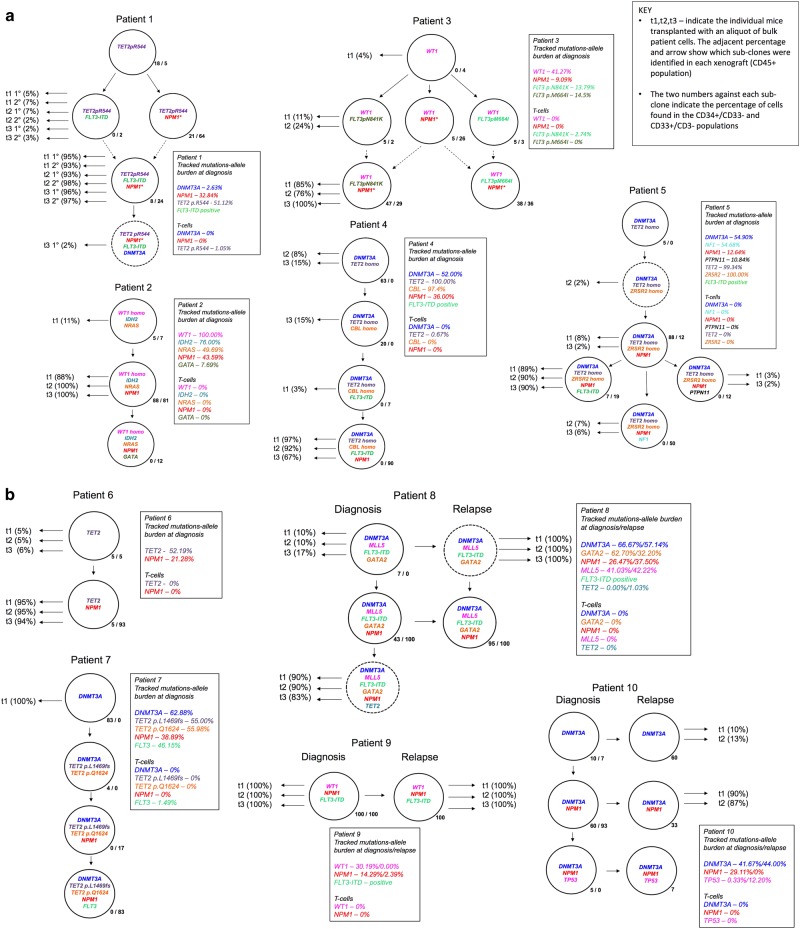
Clonal phylogenies, inferred by maximum parsimony, and sub-clone genotypes in 10 patients. Genetically distinct sub-clone percentages (as a fraction of the total population) are indicated next to each clone; e.g., patient 1, most primitive sub-clone, CD34^+^/CD33^−^ first and CD33^+^/CD34^-^/CD3^−^ second percentages indicated as 18%/5%, respectively. This indicates that this sub-clone was found in 18% of the total CD34^+^/CD33^-^ cells investigated and 5% of the total CD33^+^/CD34^−^/CD3^−^cells investigated (for the relapse samples of patients 9 and 10 only bulk cells without phenotype consideration could be sorted, as the samples available were from fixed cytogenetic preparations; the sub-clone is shown as a single percentage). Those sub-clones that grew in mice are indicated with horizontal black arrows. t1-3 (%). T, transplant. 1-3 individual mice. % fraction of human cells in mouse bone marrow. Sub-clone denoted by dotted circle is below detection limit in diagnostic sample but present in mouse transplant read-out. Dotted arrows lines between sub-clones (case #1 and #3) indicates alternative clonal phylogenies. In case #3, there are 4 possible equally parsimonious phylogenetic trees (details in Supplementary Information Figs. 5 and 6). Further details on each of the individual 10 patients’ clonal analyses are given in Supplementary Information

Figure 1 summarises the data from all ten cases (see Supplementary Information for more detailed data). This includes an identifier (t = transplant) of sub-clones that successfully transplanted into mice (t1, t2 and t3 refer to individually transplanted mice using diagnostic material from each patient). The phylogenetic or sub-clonal architectures inferred are relatively simple and either linear or branching (three patients). The analyses are relatively insensitive however with minor clones below 5% being difficult to detect. It is very likely that we are significantly under-estimating clonal complexity and will have missed minor sub-clones that could be clinically relevant, emerging at relapse [23].

In two patients (#1 and #3; Fig. 1), there were more than one equally parsimonious phylogenetic trees (illustrated by alternative dotted lines connecting sub-clones). We depict all equally parsimonious trees for patients #1 and #3 in Supplementary Figs. 5 and 6.

The number of identifiable sub-clones varied from one to six. In four patients (#4, #5, #7 and #8) the small, putative stem cell CD34^+^/CD33^−^ fractions contained a genetic sub-clone that was not discernible in the large CD33^+^ blast population. These cells had fewer mutations, lacked *NPM1*c mutation and could represent pre-leukaemic cells [8, 9].

We did not detect *DNM3TA* or other putative founder mutations in the T cells by single cell analysis. However, in most cases reported by Shlush et al. [24], the mutant *DNM3TA* allele frequency in T cells was low and so could have been missed in our samples in which only a maximum of 48 single T cells were assayed. In the total or bulk population of *NPM1c* AML-derived T cells that were subject to targeted sequencing in our series of patients, the calculated allele frequency for *DNM3TA* mutations and other putative driver mutations in AML cells ranged from 0.64 to 4.35% in the T cell population.

*NPM1c* mutations were always preceded by mutations previously considered as possible founders; *DNMT3A*, *IDH2*, *WT1*, *TET2,* as well as some additional mutations that are less well validated as early events in *NPM1c* AML including *NRAS*, *ZRSR2* and *CBL*. *FLT3* mutations and *FLT3*^*−*^*ITDs* were found to occur both before and after the acquisition of *NPM1c* but were always sub-clonal to putative founder mutations.

### Match relapsed cases

In the three *NPM1*c AMLs with matched relapse samples (#8, 9, 10), we found high levels of *NPM1*c sub-clones in the CD34^+^/CD33^−^ population at diagnosis ranging from 43–100%. In the single case in which the CD34^+^/CD33^−^ population could be assessed at relapse (patient #8), the size of the *NPM1*c sub-clone had increased from 43 to 95%. In these AMLs it was also possible to identify sub-clones at relapse or in the mice after transplant of the diagnostic or relapsed material that had acquired more mutations in addition to those found in the major clone at diagnosis (in #9, #10; see Table 1). Some of these mutations could not be tracked by Q-PCR but were identified by direct sequencing (Table 1). Patient #10 had two *FLT3* sub-clonal mutations (detected by sequencing), one at diagnosis (10.03%) rising to 41.64% whilst the other was only detected at relapse (41.16%). Neither of these *FLT3* mutations could be tracked, so they do not appear in patient #10 clonal structure (Fig. 1).

### Reiterative mutations

Reiterated mutations in individual driver genes were identified in some cases. In patient #3, the two distinctive *FLT3* mutations were segregated in distinctive sub-clones. Similarly, in patient #1, the two distinctive *TET2* mutations were present in separate (minor/major) sub-clones. In contrast, in patient #7 the two *TET2* mutations were in the same sub-clone and probably bi-allelic. Phylogenetic architectures suggested that *NPM1*c mutations may also have been reiterative in some cases, for example with patient #3 (and possibly patient #1) but the invariant nature of this mutation makes this more ambiguous.

### Stem cell read-outs in transplants

The single cell genetics of regenerated leukaemias in mice (see t1,t2,t3 % in Fig. 1) allowed us to infer the sub-clonal origins of leukaemias and hence the genetic composition and its variation in the stem or leukaemia propagating cell compartment of these AML. The clonal read-outs in the transplants were diverse but some patterns emerged.

In eight cases (patients #1, #2, #3, #4, #5, #6, #8 at diagnosis, #10 at relapse) two to five sub-clones present in the diagnostic sample regenerated in the mice. However in each case, one sub-clone was dominant, proportionally and this sub-clone always contained *NPM1c*.

In one patient (#7), only one sub-clone was present at low levels (0.39% CD34^+^ cells) in a single mouse and, surprisingly, this corresponded to the most ancestral sub-clone in the diagnostic sample which had *DNMT3A* as its sole identifier mutation. These are most likely pre-leukaemic cells. In patient #8, two sub-clones read-out in mice from the diagnostic sample. The dominant or largest sub-clone in all three mice harboured not only a *NPM1c* but also a *TET2* mutation; this clone was below the detection limit in the diagnostic sample itself (indicated by dotted circle in Fig. 1). The relapse sample from patient #8 contained only one *NPM1c* sub-clone corresponding to the major sub-clone seen at diagnosis. However, in the transplant of this sample, a *NPM1c*-negative sub-clone, ancestral to the relapse sub-clone, represented 100% of the regenerated leukaemia.

Finally, in patient #9, there was only one clone discernible both at diagnosis and relapse and this clone read-out consistently in transplants of diagnostic and relapse samples.

## Discussion

These single cell data provide definitive identification of clonal architectures and preferential order of mutations, furthering endorse the concept of sub-clonal complexity in myeloid leukaemia [7, 14–16]. However, the current limits of single cell screening means that we will have under-estimated the extent of sub-clonal genetic diversity that can be revealed by ultra-deep sequencing [25] and by new technologies that allow interrogation of thousands of cells [26]. This has implications for clonal architecture and phylogeny. For example, in diagnostic samples from several patients (#2, #3, #4, #8, #9), the sub-clone with the most simple genetic composition at the base of the phylogenetic tree harboured more than one mutation. The phylogenetic structure is therefore likely to have missed earlier, sequential (pre-leukaemic) clones [8].

Different driver mutations have epistatic or synergistic functional impacts in AML [7, 22, 27] and the order of mutation accrual may impact on stem/progenitor cell function and clinical features [28]. Our data provides direct evidence that *NPM1c* mutation is a sub-clonal and therefore secondary mutation rather than a truncal or initiating lesion, as previously suggested [29]. This concurs with the observations of Shlush et al. [9] who found (in ten patients with AML) that *DNMT3A* mutations in AML were present in differentiation competent haemopoietic stem cells and putative pre-leukaemic clones. *NPM1* mutations, in contrast, were absent from such cells but present in blasts cells with a myeloid progenitor cell phenotype presumed to be descended from the *DNMT3A* mutant clones. Similarly, Corces-Zimmerman et al. [30] found that *NPM1c* mutations were absent in purified haemopoietic stem cells, in contrast to putative founder mutations including *DNMT3A, IDH1, IDH2* and *ASXL1*. In cases of AML analysed at the single cell level, Jan et al. [8] (one case) and Quek et al. [16] (three cases) documented that *NPM1c* was sub-clonal or secondary to *TET2* mutations. However, Quek et al. [16] also identified, in two cases, very rare CD34^+^ cells that had *NPM1* mutations but not other mutations found in the bulk leukaemic cells raising the possibility that *NPM1* might occasionally be a founder mutation in pre-leukaemic cells. The preservation of diagnostic *DNMT3A* but not *NPM1c* mutations in remission [9, 31] and in a small minority of relapses is also commensurate with the predominantly secondary, sub-clonal nature of *NPM1c* [32, 33]. As is the presence of *DNMT3A* and *TET2* but not *NPM1c* mutations in covert pre-malignant clones of normal, ageing adults [34].

A preferential order of mutation may reflect genetic network or cell context dependencies. *NPM1c* (and *FLT3* mutations) might be potent drivers only when arising in myeloid progenitor cells with enhanced self-renewal provided by mutations in epigenetic mutations such as *DNM3TA* or *TET2*.

In the bulk blast cell population, *DNMT3A* and *NPM1c* mutations were present at similar high allele burden suggesting these were concurrent in the same cells [9]. In another study however, *NPM1c* allele burden was consistently less than that of other drivers including *DNMT3A* commensurate with a sub-clonal origin [19]. In our series, the allele burden for *NPM1c* was consistently less than that of other putative founder mutations including *DNMT3A*, *TET2* and *IDH2* (Fig. 1). The existence of clones ancestral to those with *NPM1c* mutations was clearly evident (in 8/10 cases) in the minor population sub-fractionated as CD34^+^/CD33^-^. This again accords with the data of Shlush et al. [9].

Mouse models with transgene or knock-in *NPM1c* have been developed to assess the role of *NPM1* in leukaemogenesis [35]. By itself *NPM1* expressed in haemopoietic stem cells produces a myeloproliferative disorder and a low penetrance of late occurring AML. A high frequency of AML does develop in *NPM1c* mice subjected to insertional mutagenesis [36] or in compound mutant mice with both *NPM1c* and *FLT3*-*ITD* [37, 38]. These modelling data testify to the functional impact of *NPM1c* on myeloid cells and leukaemogenesis but underscore that it is, at best, a weak initiating or founder lesion for AML.

The order of mutations and their position in the phylogenic tree is relevant to the selection of mutated gene for targeted therapy [4]. In the cases of *NPM1c*^+^ AML, the phylogenetic studies highlight *DNMT3A* and *TET2* as truncal mutations as reported previously [8, 9]. Effective therapeutic targeting of either *NPM1c* or *FLT3* mutations might be expected to debulk the leukaemia but with only transient benefit. However, persistence or increase of MRD in AML via detection of *NPM1c* transcripts is strongly predictive of relapse [39] and in the great majority (>95%) of cases of *NPM1c*^+^ AML that relapse, the relapsing clone is *NPM1c*^+^ [40]. In contrast, persistence of founder mutations (*DNMT3A, TET2, ASXL1*) or pre-leukaemic clones, is not predictive of relapse [41] This reflects the strong driver status of *NPM1c* mutations and the malignant potential of *NPM1c* sub-clones which is likely contingent upon the genetic background of founder (truncal) mutations (i.e., by epistasis) and additional co-existing sub-clonal mutations (e.g., in *FLT3*). Effective targeting of *NPM1c* could, therefore, be very beneficial in restraining progression of disease.

There was evidence for reiterated driver mutations in sub-clones of several cases in this study. This has been described before in ALL [3] and other cancers [42]. Mutations that are highly recurrent between patients with a sub-type of leukaemia (or any cancer) might be expected to occur more than once within a leukaemia from single patients. Functionally, this could reflect either the fitness advantage of bi-allelic mutations of the same gene in the same cells or convergent evolution of sub-clones contingent upon prevalent selective pressures or preferential, epistatic partnership with earlier, common mutations [43].

A comparison of clonal structures in three cases of matched diagnosis and relapse samples (#8, #9, #10) allowed us to infer the possible sub-clonal origins of the relapses. In one patient (#9), there was only one clone detectable at diagnosis and that same clone was the only clone observed at relapse. In case #8, the single relapse detected corresponded to one of two clones present at diagnosis. However, sequencing also revealed a *TET2* mutation at low allele burden (1.03%) at relapse. The allele burden for this mutation at diagnosis was undetectable. However, when the diagnostic sample was transplanted into mice, a sub-clone with that ‘relapse’ *TET2* mutation was the dominant clone (refer to Fig. 1 for case #8).

In patient #10, there were three sub-clones at diagnosis and all three were present in the relapse sample. These data raise the possibility that relapse in AML is not necessarily monoclonal and this should be further explored as it has important implications for the basis of drug resistance.

Xeno-transplant read-outs depend upon the genetic background of the immuno-deficient mice [15] and may not faithfully reflect the true diversity of propagating cells in AML. Furthermore, we made no attempt to titrate leukaemia propagating activity by varying the number of cells transplanted or by serial transplantation (except in patient #1). We note however that replicate mice provide very similar read-outs which suggest intrinsic, functional properties of AML sub-clones are being registered. The only conclusion we wish to draw from these limited transplant experiments is that multiple sub-clones from individual patients transplant indicating, as we showed previously for ALL [3] and glioblastoma [44], that individual leukaemia’s contain several, genetically distinct cells with self-renewing or leukaemia propagating activity. These cells will provide a diverse pool of cells distributed throughout the phylogenetic tree and from which relapse or drug resistance can emerge as recently demonstrated by Shlush et al. [24]. As such they function as cellular units of evolutionary selection [45, 46]. However, sub-clones have variable repopulating capacity [47] and as previously reported in AML [15], one *NPM1c* sub-clone dominated leukaemia regeneration in mice. This may reflect the increased malignant potential of this sub-clone and the contribution of *NPM1c*^+^ cells to relapse in >95% of cases [40]. In all our six cases where the diagnostic clone had both *NPM1c* and *FLT3* ITD or *FLT3* mutations, the dominant sub-clone in transplant readouts had both mutations. Competitiveness of sub-clones with this genotype in a xenotransplant context might be relevant to the very poor prognosis of AML cases that harbour a combination of mutants in *DNMT3A*, *NPM1c* and *FLT3* [7].

## Supplementary information

Supplementary Information

Supplementary Figures
