# Supplementary material for: Single cell analysis of clonal architecture in acute myeloid leukaemia
Source: Leukemia. 2018 Dec 19;33(5):1113–23. doi: 10.1038/s41375-018-0319-2 (PMC6451634; doi:10.1038/s41375-018-0319-2)
Supplement: Supplementary file 1 — Supplementary Information [file 41375_2018_319_MOESM1_ESM.docx]

**SUPPLEMENTARY INFORMATION**

**Single cell analysis of clonal architecture in acute myeloid leukaemia**

**Potter N, Miraki-Moud F, Ermini L, Titley I, Vijayaraghavan G,**

**Papaemmanuil E, Campbell P, Gribben J, Taussig D, Greaves M**

**Details of each patient’s clonal architecture and transplants (= supplementary to Figure 1 legend)**

***Patient 1****.*  In addition to the mutations identified and tracked in this leukaemia, a further TET2pC1271fs mutation was found with an allele burden of 41.09% at diagnosis but could not be tracked by Q-PCR (T-cell allele burden 0%). In the diagnostic sample 26 CD34+/CD33- cells and 161 CD33+/CD34-/CD3- cells were screened. 53% and 5% of CD34+/CD33- and CD33+/CD34-/CD3- cells respectively did not harbour any tracked mutations. The bulk diagnostic cells did engraft in three mice (t1 - 97.3% human CD45+ engraftment, t2 - 98.5% human CD45+ engraftment and t3 - 95.5% human CD45+ engraftment; 310, 315 and 248 single cells were analysed respectively). Serial secondary transplants were also successful (t1 - 89.7% human CD45+ engraftment, t2- 99.4% human CD45+ engraftment and t3 - 99.6% human CD45+ engraftment; 309, 301 and 314 single cells were analysed respectively). The *DNMT3A* mutation found at low level by sequencing the diagnostic sample was only found by single cell analysis in primary transplant mouse 3 at 2%.

***Patient 2****.*  In the diagnostic sample 42 CD34+/CD33- cells and 168 CD33+/CD34-/CD3- cells were screened. 7% and 0% of CD34+/CD33- and CD33+/CD34-/CD3- cells respectively did not harbour any tracked mutations. The bulk diagnostic cells engrafted in three mice at low levels (t1 – 6.04% human CD45+ engraftment, t2 – 5.02% human CD45+ engraftment and t3 – 9.99% human CD45+ engraftment; 321, 281 and 311 single cells were analysed respectively).

***Patient 3****.* In the diagnostic sample 42 CD34+/CD33- cells and 167 CD33+/CD34-/CD3- cells were screened. 0% of CD34+/CD33- and CD33+/CD34-/CD3- cells did not harbour any tracked mutations. The bulk diagnostic cells did engraft in three mice (t1 - 37.9% human CD45+ engraftment, t2 - 44.3% human CD45+ engraftment and t3- 62.1% human CD45+ engraftment; 297, 323 and 329 single cells were analysed respectively).

***Patient 4****.* In the diagnostic sample 42 CD34+/CD33- cells and 163 CD33+/CD34-/CD3- cells were screened. 17% and 3% of CD34+/CD33- and CD33+/CD34-/CD3- cells respectively did not harbour any tracked mutations. The bulk diagnostic cells engrafted in three mice; in two mice the engraftment was at low levels (t1 – 76.9% human CD45+ engraftment, t2 – 4.9% human CD45+ engraftment and t3 – 0.32% human CD45+ engraftment; 325, 324 and 319 single cells were analysed respectively).

***Patient 5****.* In the diagnostic sample 42 CD34+/CD33- cells and 163 CD33+/CD34-/CD3- cells were screened. 0% and 7% of CD34+/CD33- and CD33+/CD34-/CD3- cells respectively did not harbour any tracked mutations. The bulk diagnostic cells engrafted in three mice (t1 - 79.5% human CD45+ engraftment, t2 - 68.8% human CD45+ engraftment and t3 - 76.6% human CD45+ engraftment; 321, 396 and 322 single cells were analysed respectively). The sub-clone harbouring *DNMT3A, TET2 and ZRSR2* mutations alone was only found by single cell analysis in primary transplant mouse 2 at 2%.

***Patient 6***. In the diagnostic sample 42 CD34+/CD33- cells and 165 CD33+/CD34-/CD3- cells were screened. 90% and 2% of CD34+/CD33- and CD33+/CD34-/CD3- cells respectively did not harbour any tracked mutations. In addition to the mutations identified and tracked in this leukaemia, a further *DNMT3A* p.T691fs mutation was found with an allele burden of 39.58% at diagnosis but could not be tracked by Q-PCR. Furthermore, this mutation was found in the T cell population by targeted sequencing at 4.35%. The bulk diagnostic cells engrafted in three mice (t1 - 95.9% human CD45+ engraftment, t2 - 97.4% human CD45+ engraftment and t3 - 96.9% human CD45+ engraftment; 243, 315 and 239 single cells were analysed respectively).

***Patient 7***. In addition to the mutations identified and tracked in this leukaemia, a further *CTNNA1* mutation was found with an allele burden of 33.33% at diagnosis but could not be tracked by Q-PCR (T-cell allele burden 0%). In the diagnostic sample 40 CD34+/CD33- cells and 164 CD33+/CD34-/CD3- cells were screened. 13% and 0% of CD34+/CD33- and CD33+/CD34-/CD3- cells respectively did not harbour any tracked mutations. The bulk diagnostic cells did engraft in one mouse (t1 - 0.39% human CD45+ engraftment); as the population was clearly defined and single cells could be collected; a small population of 76 cells was investigated.

***Patient 8***. In the diagnostic sample 28 CD34+/CD33- cells and 90 CD33+/CD34-/CD3- cells were screened. 50% and 0% of CD34+/CD33- and CD33+/CD3- cells respectively did not harbour any tracked mutations. In the relapse sample 42 CD34+/CD33- cells and 168 CD33+/CD34-/CD3- cells were screened. 5% and 0% of CD34+/CD33- and CD33+/CD34-/CD3- cells respectively did not harbour any tracked mutations. The bulk diagnostic cells did engraft in three mice (t1 – 79.1% engraftment, t2 – 48.4% engraftment and t3- 54.5% engraftment; 331, 329 and 328 single cells were analysed respectively). The bulk relapse cells also engrafted in three mice (t1 – 88.1% human CD45+ engraftment, t2 – 80.6% human CD45+ engraftment and t3- 89.4% human CD45+ engraftment; 322, 310 and 307 single cells were analysed respectively).

***Patient 9***. In addition to the mutations identified and tracked in this leukaemia, two further mutations could not be tracked by Q-PCR. Mutation *MLL3pG824S* with an allele burden of 10.4% and 11.6% at diagnosis and relapse respectively and mutation, *UTYpA376D,* with an allele burden of 14.81% at relapse only (both mutations showed a T-cell allele burden of 0%). In the diagnostic sample 41 CD34+/CD33- cells and 167 CD33+/CD34-/CD3- cells were screened. 0% of CD34+/CD33- and CD33+/CD34-/CD3- cells did not harbour any tracked mutations. 168 fixed bulk cells were screened at relapse (only bulk cells without phenotype consideration could be sorted, as the samples available were from fixed cytogenetic preparations; this is indicated by the single sub-clone percentage). Both diagnostic and relapse leukaemias did engraft. However, the relapse sample showed a low level of engraftment (Diagnosis: t1 - 13.1% human CD45+ engraftment, t2 - 10.1% human CD45+ engraftment and t3 - 61.6% human CD45+ engraftment; 303, 315 and 319 single cells were analysed respectively. Relapse: t1 - 2.12% human CD45+ engraftment, t2 - 0.098% human CD45+ engraftment and t3 - 0.12% human CD45+ engraftment; 222, 65 and 70 single cells were analysed respectively).

***Patient 10***. In addition to the mutations identified and tracked in this leukaemia, two further *FLT3* mutations were also found but could not be tracked by Q-PCR; mutant *FLT3pD835G* with an allele burden of 10.03% at diagnosis rising to 41.64% at relapse. The second mutation (*FLT3pD835N*) could only be detected at relapse with a burden of 41.16% (both mutations showed a T-cell allele burden of 0%). In the diagnostic sample 41 CD34+/CD33- cells and 159 CD33+/CD34-/CD3- cells were screened. 25% and 0% of CD34+/CD33- and CD33+/CD34-/CD3- cells respectively did not harbour any tracked mutations. 165 fixed bulk cells were screened at relapse (only bulk cells without phenotype consideration could be sorted, as the samples available were from fixed cytogenetic preparations; this is indicated by the single sub-clone percentage). Engraftment was not successful when diagnostic bulk cells were transplanted but low level engraftment was achieved from relapse samples in 2 mice (t1 - 4.75% human CD45+ engraftment and t2 - 7.51% human CD45+ engraftment; 303 and 310 single cells were analysed respectively).

**Bulk FACS cell sorting**

All antibodies were obtained from BD Biosciences. Samples were stained with combinations of phycoerythrin (PE)–conjugated anti-human CD33 (catalogue no. 555450), fluorescein isothiocyanate (FITC)–conjugated anti-human CD3 (catalogue no. 555332), and allophycocyanin (APC)–conjugated anti-human CD34 (catalogue no. 555824), phycoerythrin (PE)–conjugated anti-human CD45 (catalogue no. 555483), or PE-CY7–conjugated anti-mouse CD45 (catalogue no. 553082) antibodies before resuspension in a 4,6 diamidino-2-phenylindole (DAPI) containing solution of 2% fetal calf serum (FCS) with phosphate buffered solution (PBS). Analysis and sorting was performed on a BDFACSAriaIIμ. Gates were set up to exclude nonviable cells and debris. Bulk cell sorting was performed after cell staining to isolate populations of interest for further investigation. Supplementary Figure 1 provides an example of the gated and sorted populations for peripheral blood T-cells (CD3^+^/CD33^-^) (as a control), mononuclear blast cells (CD33^+^/CD3^-^) and putative stem cell populations (CD34^+^/CD33^-^). Note that the CD33+ sorted population was less than 1% CD34+ in all patients and is therefore referred to as CD34-. Supplementary Figures 2 and 3 provide examples of xenograft FACS (BD LSR Fortessa) analysis for two patients; one with low engraftment (Figure 2) and a second with high engraftment (Figure 3).

**Library preparation and sequencing**

Custom RNA baits were designed complementary to all coding exons of 111 AML and cancer genes as per manufacturers’ guidelines (SureSelect, Agilent, UK). Whole genome amplification (WGA) with Phi 29 (Qiagen, UK) was performed to increase DNA quantity as well as to normalize concentrations. A total of 125μl of 40ng/μl of WGA DNA was fragmented to an average insert size of 145bp (75I300) and subjected to Illumina DNA sequencing library preparation using the Bravo automated liquid handling platform. Libraries were prepared and hybridized to custom RNA baits following the Agilent SureSelect 8 protocol and sequenced on the HiSeq machine using the 100 base pair paired end protocol.

**Sequencing data alignment**

Raw sequence data were aligned to the human genome (NCBI build 37) using BWA4. Unmapped reads, PCR duplicates and reads mapping to regions outside of the target region (merged exonic regions + 10bp either side of each exon) were excluded from analysis. Bedtools® coverage v2.15.05 was subsequently used to determine the coverage depth at each base. Genes with median target coverage < 20x were removed from the analysis on a case by case basis.

**Bioinformatics analysis**

The majority of gene mutations in *NPM1*^+^ AML are single base pair substitutions, but small indels and complex events such as the FLT3 internal tandem duplication (ITD) are common. In the present study, base substitutions were identified using two parallel bioinformatics approaches. Single base, somatic substitutions were called independently in each sample using an in house algorithm CaVEMan: Cancer Variants through Expectation Maximisation. https://github.com/cancerit/CaVEMan. The algorithm compares sequence data from each tumour sample to an unrelated normal sample and calculates a mutation probability at each base pair position locus. A number of post processing filters were applied to improve specificity (see below ‘Filters applied to sequencing data’).

The second approach used an algorithm that incorporates information from multiple unrelated samples and prior knowledge to generate local error estimates and call mutations in unmatched targeted resequencing data https://github.com/mg14/deepSNV. For hotspot mutations Samtools mpileup was employed to specifically interrogate mutations in known hot spot regions.

**Filters applied to sequencing data**

Filters applied to targeted capture data required that:

1. At least a third of the alleles containing the mutant must have base quality>=25.
2. If mutant allele coverage >=10, there must be a mutant allele of at least base quality 20 in the middle 3rd of a read. If mutant allele coverage is <10, a mutant allele of at least base quality 20 in the first 2/3 of a read is acceptable.
3. The mutation position is marked by <3 reads in any sample in the unmatched normal panel.
4. If the mean base quality is <20 then less than 96% of mutation carrying reads are in one direction.
5. Variants were cross referenced with approximately 300 unmatched normal samples sequenced internally to identify calls coinciding with high error rate loci.
6. Previously reported bona fide somatic variants presenting in the unmatched normal panel were not filtered out from the dataset.
7. Variants presenting in the patients matched control CD3^+^/CD33^-^ cell population at a higher frequency than 5% were considered to be a constitutional alteration and ignored.

**Selection criteria for calling non-coding indels**

1. ‘SUMIMS’ score (sum of the mapping scores of the reads used as anchors)>=200.
2. ‘Previously Rejected Score (PRS) is =0.
3. Bidirectional (evidence in both read directions (forward and reverse) in Pindel or BWA reads).
4. Variant allele is not a unit within a homopolymer track presenting with variant allele fraction <8%.
5. Variants did not present in approximately 300 unmatched normal samples and did not have a COSMIC ID with confirmed somatic status in the literature.
6. Artefactual indels occur at recurrent loci across multiple samples, often as a consequence of highly repetitive sequence. To ensure that such variants were not retained, we interrogated for recurrently rejected Pindel calls. All variants were visually inspected prior to removal. Regions enriched for GC content and low target coverage were manually reviewed (i.e. *CEBPA*, *SRSF2*). Additionally, for *FLT3*-*ITD* detection we developed custom analysis script that performs a localized query for reads consistent with an inverted tandem duplication within the FLT3 locus. Visual inspection using visualisation software (Gbrowse®) was performed of all variants in the targeted gene screen data set after applying these filters.

**Small insertions and deletions including FLT3-ITD**

Small somatic insertions and deletions (indels) were identified using a modified version of Pindel8. Post processing filters were applied as previously described ^1, 2^. A number of steps were taken to improve specificity for calling non coding indels (see below ‘Selection criteria for calling non-coding indels’).

It has been previously reported that the size and complexity of an indel affects mapping efficiency of reads reporting indel variants resulting in under estimates of the absolute variant allele fraction for the mutant alleles. Correction for this effect is described under ‘Correction of variant allele fraction’ below.

**Correction of variant allele fraction**

To correct for this effect, the burden of each indel in all related samples was determined by counting both mapped and unmapped reads within the repeat range of the indel. This was achieved by generating a variant haplotype containing the reported indel followed by BLAT realignment of unmapped singleton reads anchored by a mapped read within the expected region of the indel (using average library size for sample as a reference). This was followed by PCR duplicate removal. All newly mapped reads reporting the indel were then added to the paired mapped reads, and unique allele counts for each indel were generated ^3^. To expand on this further, a comparison was made between the mutant allele burdens and number of mutant positive single cells (Supplementary Figure 4). Overall the data was comparable but the allele burdens generate for *NPM1* mutations underestimated the number of positive cells in all patients.

Alterations found for each patient were validated by Q-PCR using in-house designed allelic discrimination assays and Applied Biosystems standard methods and protocols (Table 3 in main text); these assays were subsequently used for single cell analysis. If it was not possible to design a Q-PCR assay for a specific mutation, the mutations were validated by traditional RT-PCR and Sanger Sequencing but not tracked in the single cell analysis.

**Xeno-transplantation**

All work was performed in accordance with Home Office regulations after approval by the Queen Mary University of London Animal Welfare and Ethical Review Body.

Mice with successful grafts (when >2% of blood leukocytes are human leukaemia cells) were sacrificed and bone marrow cells harvested for further investigation. (Purified leukaemia cells were transplanted into secondary recipients).

Mice with undetectable/minimal (<2%) grafts at 12-14 weeks (based on analysis of peripheral blood) were re-bled at later time points. Some *NPM1*c AML samples proliferate relatively slowly within NSG mice. These mice were sacrificed at 18-26 weeks once repeat blood analysis showed greater than 2% leukaemia cells. Beyond 18 weeks, secondary transplants were deemed unnecessary, as leukaemia cells had demonstrated long-term repopulating abilities; a key feature of true leukaemic stem cells. In those cases where engraftment was successful the human CD45+ population was sorted for single cell analysis.

**Serial transplantation**

Serial transplantation was completed for one case. Bone marrow cells from mice transplanted with fractions of AML were stained with PE-C57-conjugated anti–mouse CD45 antibody and PE-conjugated anti–human CD45 antibody before resuspension in a DAPI-containing solution of 2% FCS with PBS. Human CD45+ cells were sorted on a BDFACSAriaIIμ before transplantation into secondary mice. In mice where engraftment of leukemia was high, bone marrow cells were transplanted without cell sorting.

**Single cell sorting and multiplex Q-PCR analysis**

A cell was deemed to be positive for a single nucleotide variant if the Q-PCR cycle threshold (C_T_) value was below 25. The presence or absence of the signal from the probe complementary to the wild-type sequence determined heterozygous or homozygous mutations respectively.

To estimate the error rate of each assay a control experiment consisting of 48 cord blood cells was completed for each patient specific multiplexed experiment. Assay error rates in these experiments were 0. Data from single cells that were removed from the Q-PCR analysis included those wells that showed no data (no cell) and those wells in which all *ß2M* assays did not have a strong signal (<25 C_T_). On average this accounted for the removal of ~7% of data points. Only mutational spectra occurring in more than one cell was included in the phylogenetic analysis.

**Maximum parsimony**

Heuristic searches were performed with a series of 1 million random additional clones and tree branch-swapping using a bisection-reconnection (TBR) algorithm. All characters weighted equally, and state graphs and step matrices were used to assign equal costs to each character state transition in different genes. The cost assigned for each transition is linear and results from the equation yi = 2xi. The normal clone (according to the alterations interrogated) found in each case was assumed to be the ancestral clone and included in the analysis as the root of the tree/trees. All parsimony analyses were performed using the computer software PAUP* version 4.0b10 for Linux ^4^. Trees were visualized using Dendroscope Soft-ware version 3 ^5^. Supplementary Figures 5 and 6 indicate the results of maximum parsimony searches in patients #1 and #3 with branching sub-clonal architectures and/or reiterative mutations.

**REFERENCES**

1. Potter NE, Ermini L, Papaemmanuil E, Cazzaniga G, Vijayaraghavan G, Titley I*, et al.* Single-cell mutational profiling and clonal phylogeny in cancer. *Genome Res* 2013 Dec; **23**(12)**:** 2115-2125.

2. Kreso A, Dick JE. Evolution of the cancer stem cell model. *Cell Stem Cell* 2014 Mar 6; **14**(3)**:** 275-291.

3. Papaemmanuil E, Gerstung M, Bullinger L, Gaidzik VI, Paschka P, Roberts ND*, et al.* Genomic Classification and Prognosis in Acute Myeloid Leukemia. *N Engl J Med* 2016 Jun 9; **374**(23)**:** 2209-2221.

4. Holder MT, Lewis PO, Swofford DL, Larget B. Hastings ratio of the LOCAL proposal used in Bayesian phylogenetics. *Syst Biol* 2005 Dec; **54**(6)**:** 961-965.

5. Huson DH, Scornavacca C. Dendroscope 3: an interactive tool for rooted phylogenetic trees and networks. *Syst Biol* 2012 Dec 1; **61**(6)**:** 1061-1067.

**SUPPLEMENTARY FIGURE LEGENDS**

**Supplementary Figure 1**

This figure illustrates the flow cytometry sorting approach used to collect the populations of interest in this study; CD3+/CD33- cells, CD33+/CD3- and CD34+/CD33- cells. Initially DAPI and forward scatter were used to identify viable cells; Supplementary Figure 1a – region p8. Cells in p8 were then investigated for side scatter height and area to identify individual single cells; Supplementary Figure 1b – region p11. Cell aggregates have a higher side scatter area:height ratio. Positive vs negative combinations of specific antibodies (as described in materials and methods) were used to identify the populations of interest. CD3+/CD33- cells were collected from gate p9 (regions p8+p11+p9) (Supplementary Figure 1c); CD33+/CD3- were collected from gate p10 (regions p8+p11+p10) (Supplementary Figure 1c); CD34+/CD33- cells were collected from gate p4 (regions p8+p11+p2+p4) (Supplementary Figure 1d) which originated from region p2 (Supplementary Figure 1c).

**Supplementary Figures 2 and 3 (a and b)**

For each patient, NOD/SCID (Il2rg^-/-^) mice (x3) were injected intravenously with 9-10 million AML cells after T-cell depletion. The figures here illustrate the engraftment, shown by flow cytometry (BD LSR Fortessa), in three mice for a patient that engrafted at a low level (Supplementary Figure 2; patient 7) and a patient that engrafted at a high level (Supplementary Figure 3; patient 3).

For patient 7 no human cells (hCD45 positive) were identified in any mouse after 11 weeks (Supplementary Figure 2a; first bleed). At 25 weeks when the mice were sacrificed only one mouse showed engraftment in the bone marrow above 0.1% (Supplementary Figure 2b); 76 single cells were collected and screened for patient specific alterations.

For patient 3 no human cells (hCD45 positive) were identified in any mouse after 11 weeks (Supplementary Figure 3a; first bleed). At 26 weeks when the mice were sacrificed all three mice showed engraftment in the bone marrow at 37.9%, 44.3% and 62.1% (Supplementary Figure 3b); engraftment in the spleen was seen at or below 1%.

**Supplementary Figure 4a**

For each patient specific mutation the mutant allele burden (generated by targeted NGS) and the number of positive single cells are expected to have a positive relationship. An allele burden of 50% suggests that every single cell would be positive for the mutation. To confirm this relationship the generated mutant allele burden and number of mutant positive single cells were plotted for every mutation investigated. Green dots represent homozygous mutations that have an allele burden of approximately 100% and were found in almost all single cells screened. The red dots represent the *NPM1* mutations investigated in each patient; the frequency of which was significantly underestimated by targeted NGS. This is most likely due to the fact that this mutation is a 4 base pair insertion and not a SNV.

**Supplementary Figure 4b**

If both the homozygous and *NPM1* mutation data are removed from this analysis the linear trend line is as expected; only marginally rising above the expected allele burden of 50% when the same mutation was found in every cell. The majority of mutations have an allele burden of ±10%; 6/23 are outside this range.

**Supplementary Figure 5.**

Maximum parsimony analysis of clonal structure for patient #1:

*Top*: two equally parsimonious trees were identified in the bulk CD33^+^/CD3^-^ cell fraction.

*Bottom*: one phylogenetic tree only was identified in the CD34^+^/CD33^-^ fraction.

**Supplementary Figure 6.**

Maximum parsimony analysis of clonal structure for patient #3.

*Left*: four equally parsimonious trees for the bulk CD33^+^/CD34^-^ cell fraction.

*Right*: four equally parsimonious tree for the putative stem cell CD34^+^/CD33^-^ fraction.

Phylogenetic analysis is based on the detected number of sub-clones and inferred by the maximum parsimony algorithm. Leukaemic sub-clones are represented by yellow circles and the normal state is indicated by a black or grey circle; black inferred grey detected. The size of each circle is directly proportional to the number of single cells included in the sub-clone and the detected genetic markers are listed below each circle. Tree branch lengths are directly proportional to the number of evolutionary changes and the scale bar at the left bottom of each tree provides a scale for this. The number in italics at each node indicates the bootstrap value. The N label indicates the normal state, the C label indicates a tumour state.
