# Supplementary material for: Single cell analysis of clonal architecture in acute myeloid leukaemia
Source: Leukemia. 2018 Dec 19;33(5):1113–23. doi: 10.1038/s41375-018-0319-2 (PMC6451634; doi:10.1038/s41375-018-0319-2)
Supplement: Supplementary file 2 — Supplementary Figures [file 41375_2018_319_MOESM2_ESM.docx]

Supplemantary Figure 4a - Comparison of mutant allele burdens and number of mutant positive single cells

Supplementary Figure 4b - Comparison of mutant allele burdens and number of mutant positive single cells

Supplementary Figure 5

Supplementary Figure 6
